# Advances in epigenetic research of adolescent idiopathic scoliosis and congenital scoliosis

**DOI:** 10.3389/fgene.2023.1211376

**Published:** 2023-07-26

**Authors:** Duan Sun, Zihao Ding, Yong Hai, Yunzhong Cheng

**Affiliations:** Department of Orthopedic Surgery, Beijing Chao-Yang Hospital, Capital Medical University, Beijing, China

**Keywords:** epigenetics, biomarkers, signaling pathways, adolescent idiopathic scoliosis, congenital scoliosis

## Abstract

Scoliosis is a three-dimensional structural deformity of the spine; more than 80% of scoliosis has no specific pathogenesis but is understood to be closely related to genetic, hormonal, and environmental factors. In recent years, the epigenetic alterations observed in scoliosis have been analyzed in numerous studies to determine the pathogenesis and progression of this condition, however, there is currently no comprehensive review of the epigenetic factors to date. We searched PubMed, Embase, and Web of Science databases for relative studies without language and date restrictions in March 2023. Twenty-five studies were included in this review and analyzed from the four main aspects of epigenetic alteration: DNA methylation, non-coding RNAs, histone modifications, and chromatin remodeling. The relationship between DNA methylation, non-coding RNAs, and scoliosis was considerably reported in the literature, and the corresponding related signaling pathways and novel biomarkers observed in scoliosis provide insights into innovative prevention and treatment strategies. However, the role of histone modifications is rarely reported in scoliosis, and few studies have investigated the relationship between scoliosis and chromatin remodeling. Therefore, these related fields need to be further explored to elucidate the overall effects of epigenetics in scoliosis.

## Introduction

Scoliosis is a generic term that describes a three-dimensional structural deformity of the spine (Giampietro, 2012), primarily encompassing adolescent idiopathic scoliosis (AIS) (Weinstein et al., 2008), congenital scoliosis (CS) (Hedequist and Emans, 2007; Pahys and orthopedics, 2018), and neuromuscular scoliosis (NMS) (Vialle et al., 2013; Wishart et al., 2021). The pathogenesis of scoliosis has not been established but is currently considered to be associated with a variety of factors, including genetic, hormonal, and environmental factors (Qiu, 2018). Nonetheless, in 2013, Fendri et al. (2013) identified a characteristic gene expression pattern in patients with AIS and used RT-PCR to identify 145 differentially expressed genes (DEGs). Therefore, the effects of epigenetic modifications in scoliosis have attracted the attention of numerous researchers in recent years.

Epigenetics is defined as variations in gene expression that are not caused by DNA sequence alterations and are heritable in mitosis and meiosis (Delcuve et al., 2009). Key epigenetic mechanisms include DNA methylation, histone modification, non-coding RNA, and chromatin remodeling (Delcuve et al., 2009). Epigenetic alterations can regulate gene expression at various stages, including replication, transcription, and translation. Epigenetic changes are known to be involved in the development of cancer, neurodegenerative pathologies, and autoimmune diseases (Nebbioso et al., 2018; Zhang et al., 2020). Current research on AIS has indicated that genetic variations only explain approximately 2%–3% of the causative factors (Peng et al., 2020), which implies the involvement of other factors, such as epigenetic changes may play an important role in scoliosis. In this review, we summarize the current research on the relationship between epigenetics and the development of scoliosis; additionally, based on the four main mechanisms of epigenetic variation, we categorized the current studies and extracted the key changes, as well as the related mechanism hypothesis and regulation-pathways.

**FIGURE 1:**
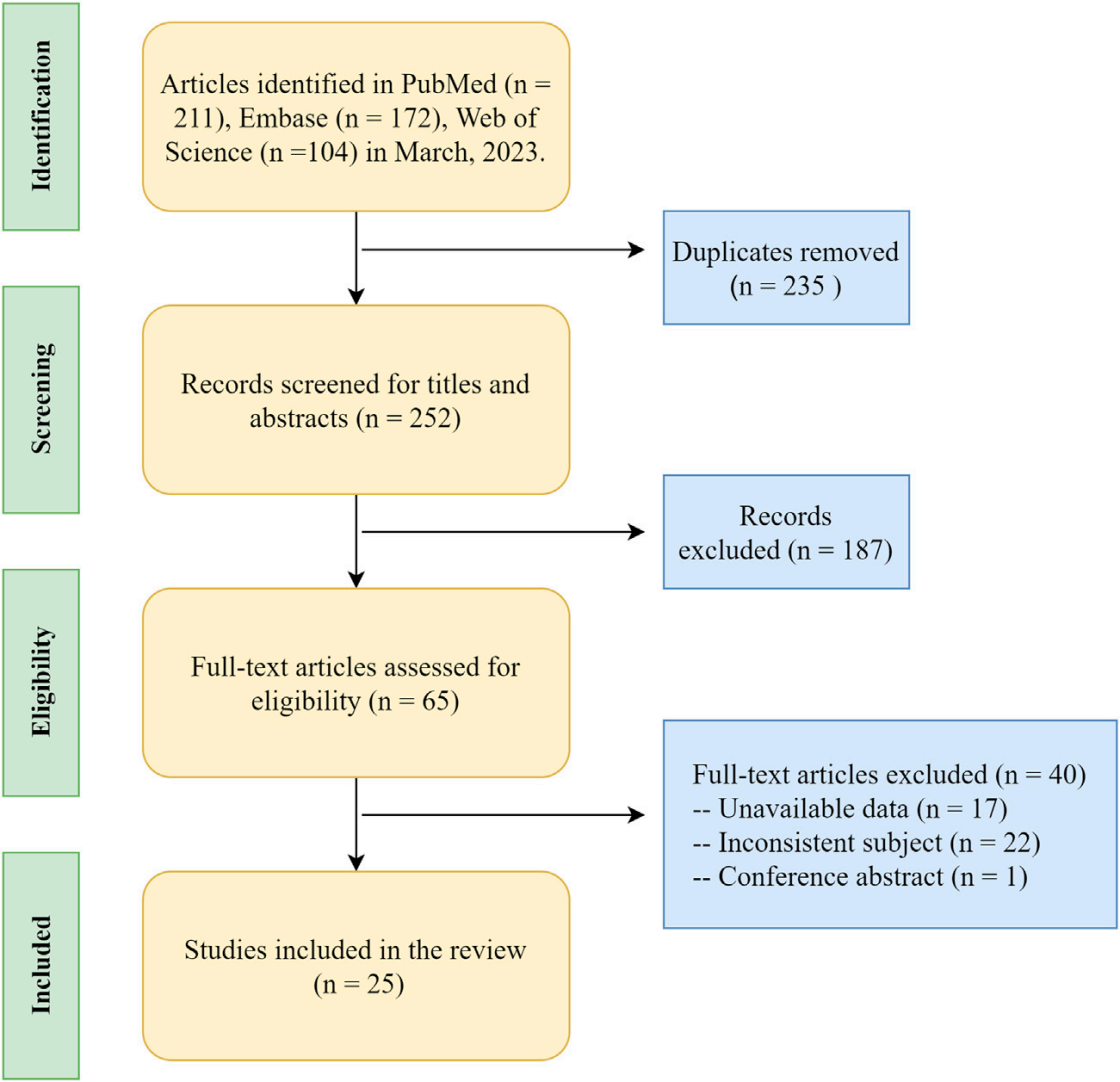
The literature search process on epigenetic research of scoliosis.

In this review, the terms “scoliosis,” “epigenetics,” “DNA methylation,” “histone modification,” “chromatin remodeling,” and “non-coding RNA” were used as keywords to find relative articles in PubMed, Embase, and Web of Science databases. This search was conducted until March 2023. The inclusion criteria were as follows: 1) clinical or basic research relevant to the above keywords; 2) papers published in journals with no language restrictions. The exclusion criteria were as follows: i) papers without full-text availability; ii) research with low relevance to the topic; iii) research with an unclear description of the original text or a relatively insufficient evidence-based rating. A total of 487 relevant papers were searched, and 25 papers were finally included in this review according to the selection criteria. Ten articles were related to DNA methylation, 13 related to noncoding RNA, 2 articles introduced histone modification, and we did not find article focused on the relationship between chromatin remodeling and scoliosis. The source of samples in each article were summarized in Table 1, 2. The literature search process is detailed in Figure 1.

**TABLE 1:** The relationship between DNA methylation and scoliosis.

Author	Year	Subject	Number(n)	Sample	Differentially methylated regions (DMRs)
Mao	2018	AIS vs. Control	50 vs. 50	Blood	A higher methylation level of COMP promoter in AIS patients
Meng	2018	AIS progression vs. AIS non-progression	50 vs. 42	Blood	A lower methylation level of the Hg19 site in the AIS progression group
Shi	2018	AIS vs. Control	50 vs. 50	Blood	Highly methylated PITX1 gene promoter in AIS patients
Liu	2019	A female MZ twin pair with 1 AIS	1 vs. 1	Blood	313 hypermethylated DMRs and 397 hypomethylated DMRs
Shi	2020	AIS vs. Control	50 vs. 50	Blood	PCDH10 gene promoter methylation levels are elevated in AIS patients
Chmielewska	2020	IS female	29	Deep paravertebral muscles	The methylation level of ESR2 promoter 0N was significantly higher in the paravertebral muscle on the concave side
Wu	2020	CS vs. Control	50 vs. 50	Blood	Higher KAT6B gene promoter methylation in CS patients
Liu	2021	CS	4	Hemivertebrae and spinal process	343 hypomethylated DMRs and 222 hypermethylated DMRs
Carry	2021	AIS discordant twins	16	Blood	57 DMRs were detected in 6 pairs of twins with significant different Cobb angle
Janusz	2021	IS female	29	Superficial muscles and deep paravertebral muscles	Paravertebral muscle on the concave side in patients with lower Cobb angle showed lower methylation in T-DMR2

AIS: Adolescent idiopathic scoliosis; MZ: monozygotic; IS: Idiopathic scoliosis; CS: Congenital scoliosis.

**TABLE 2:** Non-coding RNA and scoliosis.

Author	Year	Group	Sample	Subject	Differential expressed non-coding RNA
Liu	2015	AIS vs. Control	Blood	mRNA, lncRNA	lncRNAs:ENST00000414894.1
					lncRNAs:TCONS 00028768
					lncRNAs:ENST00000602322.1
Jiang	2017	AIS	Paravertebral muscles	miRNA	miR-517a-3p
					miR-424-3p
Seco-Cervera	2018	FRDA vs. Control	Blood	miRNA	hsa-miR-128-3p
					hsa-miR-625-3p
					hsa-miR-130b-5p
					hsa-miR-151a-5p
					hsa-miR-330-3p
					hsa-miR-323a-3p
					hsa-miR-142-3p
García-Giménez	2018	AIS vs. Control	Blood	circRNA	miR-27a-5p
					miR-223-5p
					miR-122-5p
Zhang	2018	AIS vs. Control	Iliac bone	miRNA	miR-145
Jiang	2018	AIS	Paravertebral muscles	mRNA, lncRNA	mRNA: ADIPOQ
					lncRNA: miR675-5p
Chen	2018	VAD-CS rat	Rat embryos	Noncoding RNA	miRNAs: miR-187-5p, miR-466c-3p
					lncRNA: NONRATG027649.1 NONRATG024332.1
					circRNA: chr5_50556456_51183813, chr15_23792823_23793342
Hui	2019	AIS vs. Control	BM-MSCs	miRNA	miR-17-5p
					miR-106a-5p
					miR-106b-5p
					miR-16-5p
					miR-93-5p
					miR-15a-5p
					miR-181b-5p
Zhuang	2019	AIS vs. Control	BM-MSCs	lncRNA	lncRNA: ENST00000453347
Wang	2020	Mild AIS vs. Severe AIS vs. Control	Serum	miRNA	miR-151a-3p
Liu	2020	CS vs. Control	Blood	circRNA	hsa-circ-0006719
Ishiwata	2020	CS vs. Wister mice	Spinal samples	miRNA	miR-224-5p
Chen	2022	AIS vs. Control	Iliac bone	miRNA	miR-96-5p

AIS: Adolescent idiopathic scoliosis; CS: Congenital scoliosis; FRDA: Friedreich’s ataxia; VAD-CS: Vitamin A Deficiency-Induced Congenital Scoliosis.

## DNA methylation

DNA methylation within the human genome typically occurs on the cytosine of the CpG dinucleotide, which is often located at the starting point of gene transcription (Delcuve et al., 2009). Under typical conditions, DNA methylation presents a dynamic equilibrium (Szyf, 2007), and silencing of selected genes according to physiological needs assists in controlling gene expression homeostasis (Miranda and Jones, 2007). In pathological states, abnormal DNA methylation can lead to dysregulated gene expression, which can cause abnormal expression of critical downstream products, triggering abnormal cell proliferation or apoptosis, thereby resulting in the development of scoliosis.

### Differential methylation of key loci

Deficiencies in critical components or enzymes of spinal growth and development can lead to spinal dysplasia and corresponding development or progression of scoliosis. Cartilage oligomeric matrix protein (COMP) is an extracellular matrix component essential for cartilage growth. Some studies have demonstrated that the secretion of COMP is significantly lower in patients with AIS than in normal controls (Gerdhem et al., 2015). Mao et al. examined the methylation and expression levels of COMP at five CpG loci within the *COMP* gene from patients with AIS and healthy controls (Mao et al., 2018). The level of *COMP* promoter methylation was significantly higher in patients with AIS than that in controls, whereas COMP expression was lower in patients with AIS than that in controls; additionally, the level of this abnormal methylation exhibited a positive correlation with an early onset of AIS and a larger Cobb angle. Overall, this revealed the relationship between abnormal DNA methylation and decreased COMP expression.

In 2018, Shi et al. established that the promoter of the *PITX1* gene was hypermethylated, and the expression levels of downstream products were markedly reduced in patients with AIS compared to those in controls (Shi et al., 2018). The methylation levels at the six CpG sites on the *PITX1* promoter region exhibited a correlation with the severity of scoliosis. PITX1 is a member of the RIEG/PITX homologous frame of transcription factors (Logan and Tabin, 1999), which act as a transcriptional regulator that participates in basal level regulation of prolactin, is involved in the process of hormonally regulated changes in prolactin activity; the abnormal expression of this gene has been found in many skeletal-related disorders (Alvarado et al., 2011; Pandey et al., 2012). Wu et al. found high levels of *KAT6B* gene promoter methylation and significantly reduced gene expression of *KAT6B* in patients with CS (Wu Y. et al., 2020). Correlation analysis identified a positive correlation between increased *KAT6B* methylation and an increased Cobb angle in these patients. Related research confirmed that the *KAT6B* gene was responsible for encoding part of the histone acetyltransferase and MOZ/MORF protein complexes (Tajul-Arifin et al., 2003). The MOZ/MORF protein plays a critical role in early skeletal and neuronal cell metabolism (Desh et al., 2014); therefore, aberrant DNA methylation at this site contributes to the development of CS. Taken together, this research demonstrated that the aberrant methylation of genes that play a crucial role in bone formation and development is an essential contributor to the development and progression of scoliosis.

The level of DNA methylation at key loci can also be applied as biomarkers to predict the progression of scoliosis. In 2018, Meng et al. determined that the methylation level at the cg01374129 locus was significantly lower in the AIS progression group than in the non-progression group (Meng et al., 2018). Through regression analysis, hypomethylation at this locus could be used as an independent prognostic factor for scoliosis exacerbation. Specifically, by assessing the degree of cg01374129 methylation, a sensitivity of 76.4% and specificity of 85.6% was achieved when differentiating between patients in progressive and non-progressive scoliosis groups, thereby suggesting that DNA methylation status had strong potential for use as a novel biomarker to determine prognosis. This locus is close to the gene encoding hyaluronan synthase 2 (HAS2), which plays an essential role in promoting intervertebral disc and vertebral body development in developmental rat models. Therefore, Roughley et al. suggested that abnormal methylation at the cg01374129 locus affects the progression of scoliosis via the disrupted development of the vertebral body and disc in these patients (Roughley et al., 2011).

Scoliosis progression can also be influenced by a variety of critical products of macroscopic pathways. Shi et al. found elevated *PCDH10* gene promoter methylation and downregulated *PCDH10* expression in patients with AIS compared to those in controls (Shi et al., 2020); additionally, these increased methylation levels were positively correlated with larger Cobb angles. PCDH10 is the target protein of p53, which regulates cell migration, but is not directly involved in cartilage development (Shi et al., 2015). However, as PCDH10 is involved in immunomodulatory-related roles in the Wnt-tyrosine kinase pathway, it has been postulated that in addition to directly affecting the development of bone growth, abnormal DNA methylation may also have an impact on the progression of scoliosis via the immune pathway. Overall, the studies discussed above evaluated the relationship between DNA methylation and scoliosis at a global level by analyzing peripheral blood specimens from patients with scoliosis.

Alternatively, other studies focused on DNA methylation alterations that are specific to skeletal muscle tissue around the spine of patients with scoliosis. In 2020, Chmielewska et al. examined DNA methylation levels in deep paravertebral muscle samples taken from the convex and concave sides of patients with AIS (Chmielewska et al., 2020). The methylation of estrogen receptor 2 (*ESR2*) promoter 0N in the paravertebral muscle was significantly higher on the concave side of the patient than that on the convex side. Corresponding correlation analysis revealed that *ESR2* promoter 0N methylation variation was strongly associated with the development of AIS but was not significantly associated with the degree of curvature. This study was the first to elucidate a possible mechanism for the development of scoliosis from the perspective of aberrant local tissue DNA methylation. Janusz et al. (Janusz et al., 2021)assessed the deep paravertebral and superficial dorsal muscles of patients with idiopathic scoliosis to analyze the regulation of differentially methylated regions (T-DMRs) on the estrogen receptor 1 (*ESR1*) gene. ESR1 expression was determined to correlate with the methylation of CpGs in the T-DMR2 of the concave paravertebral muscle. The methylation of the four CpG islands in the T-DMR2 of the concave paravertebral muscle was markedly low in patients with less severe scoliosis (Cobb’s angle <70°). These results establish a correlation between aberrant local tissue methylation at key loci and the progression of scoliosis.

### Methylation level differences and pathway regulation

Differentially methylated regions (DMRs) at a range of loci often differ between patients with scoliosis and normal controls; therefore, in addition to aberrant methylation at key loci, attention has been provided to the impact of aberrant methylation sites across the genome. Since monozygotic (MZ) twins are genotypically identical but phenotypically different (Simony et al., 2016), the correlation between abnormal DNA methylation and the onset and progression of scoliosis can be more clearly explored by measuring DMRs in these twins. In 2019, Liu et al. utilized a pair of MZ twins with AIS within a study to find their DMRs, and further validate the role of these DMRs in 20 patients with AIS and controls (Liu et al., 2019). A total of 313 and 397 hypermethylated and hypomethylated DMRs, respectively, were identified in this study. The main regulation of the expression of these genes is attributed to the MAPK/PI3K-Akt pathway. Previous studies identified that the MAPK/PI3K-Akt signaling pathway is primarily associated with osteoblast differentiation and bone formation (Zhang et al., 2014; Iezaki et al., 2018). Additionally, Liu et al. reported the role of MAPK and other pathways in patients with CS (Liu et al., 2021). These DMRs control the downstream production of proteins predominantly found in the MAPK and calmodulin pathways, which are involved in the regulation of cytogenesis. Additionally, the calmodulin pathway directly regulates the osteogenesis process and affects the development of vertebral bodies in patients with scoliosis. In a study conducted by Carry et al. (Carry et al., 2021), eight pairs of MZ twins who exhibited scoliosis were enrolled. In six of these pairs of twins, large differences in Cobb angles were observed; 57 DMRs were detected within these twins, of which 28 DMRs exhibited a correlation with the degree of scoliosis. Of the DMRs, cg02477677 (within the vicinity of the *RARA* gene on chromosome 17), cg08826461 (corresponding to an unknown gene on chromosome 2), cg12922161 (within the *LOC150622* gene on chromosome 2), and cg16382077 (corresponding to an unknown gene on chromosome 7) were the four most highly correlated DMRs observed in this study. Most of the corresponding gene products were enriched in the Wnt signaling pathway, which is associated with the production of neuropeptide Y (NPY). The Wnt signaling pathway has been previously reported to be important for bone formation and remodeling (Vink et al., 2014); additionally, NPY regulates bone and energy homeostasis (Wu J. Q. et al., 2020). Aberrant DNA methylation can thus contribute to the progression of scoliosis by affecting normal skeletal growth metabolism (Table 1).

## Non-coding RNA

In recent years, an increasing number of studies have demonstrated that non-coding RNAs play an essential role in the regulation of gene expression in the development of various diseases (Vondervoort et al., 2013; Jusic et al., 2020). The most common non-coding RNAs are long non-coding RNAs (lncRNAs), microRNAs (miRNAs), and circular RNAs (circRNAs). lncRNA strands are >200 nucleotides in length and are involved in transcriptional regulation, which has been observed to affect gene expression and participate in organismal regulation (Bridges et al., 2021). miRNAs are endogenously expressed non-coding transcripts that bind and silence corresponding mRNAs, thereby regulating protein expression (Hansen et al., 2013). circRNAs are non-coding RNAs with a stable circular structure. Studies found that circRNAs are rich in attachment sites to miRNAs and can affect gene expression by regulating the level of miRNAs (Hansen et al., 2013). The stability of the circular structure in circRNAs also enables them to be used as a stable biomarker that can be readily detected in the blood. (Garcia-Gimenez et al., 2018; Zhang et al., 2018).

### lncRNA

Liu et al. revealed a series of 546 mRNAs with 139 lncRNAs differentially expressed in AIS children and controls (Liu et al., 2015). In particularly, ENST00000440778.1 was significantly under-expressed, and ENST00000414894.1, TCONS 00028768, and ENST00000602322.1 were significantly over-expressed in patients with AIS. The comparison between groups indicated that children with a high expression of ENST00000602322.1 had an early onset of AIS, while patients with low expression of ENST00000440778.1 had a high grade of Risser’s sign, and a high expression of ENST00000414894.1 conferred a Cobb angle >40°. Therefore, this study demonstrated the potential of lncRNAs as a predictor of disease.

Abnormal mesenchymal stem cell differentiation is a possible etiology for the formation of scoliosis. In 2019, Zhuang et al. identified a series of 1,483 lncRNAs that were differentially expressed in the bone marrow mesenchymal stem cells (BM-MSCs) of patients with AIS and normal controls (Zhuang et al., 2019). Specifically, ENST00000453347 was significantly under-expressed in patients with AIS. This lncRNA was named lncAIS, and the corresponding difference in expression was further verified by RT-PCR analysis of samples from patients and normal subjects. BM-MSCs with lncAIS knockdown exhibited impaired differentiation in *in vitro* cytological assays; moreover, this fraction of knocked-down cells failed to effectively form bone. This study further established that in normal humans, lncAIS interacted with NF90 to promote *HOXD8* mRNA stability, thereby enhancing *RUNX2* transcription in BM-MSCs and safeguarding the osteogenic differentiation of normal BM-MSCs. Within the AIS population, a significantly low expression of lncAIS causes impaired osteogenesis of the differentiated MSCs, resulting in the development of scoliosis (Zhuang et al., 2019).

Further, the RNA changes within the paravertebral muscle tissue around the spine are of equal concern. In 2018, Jiang et al. collected paired paravertebral muscle tissue specimens from patients with AIS on the convex and concave sides of the spine and identified a total of 40 DEGs on both sides of the paravertebral muscle (Jiang et al., 2018). Specifically, these genes were enriched in the peroxisome proliferator-activated receptor (PPAR) signaling pathway, with three mRNAs, ADIPOQ, FABP4, and MSTN, being markedly differentially expressed. Additionally, the lncRNA H19, encoded by the *H19* gene, was observed to be significantly under-expressed in the concave paravertebral muscle tissue of patients with AIS, implying that this lncRNA plays an important role in the regenerative differentiation of skeletal muscle (Dey et al., 2014; Gao et al., 2014). Further, correlation analysis demonstrated that differential expression of *ADIPOQ* and *H19* mRNA in both paravertebral muscles was associated with the early onset of AIS and high lateral convexity angle.

Studies utilizing animal models have also provided new insights. Chen et al. explored the expression and mechanism of action of the lncRNA *SULT1C2A* within a rat embryo model of vitamin A deficiency–induced scoliosis (VAD-CS) (Chen et al., 2019). *SULT1C2A* expression was determined to be downregulated, rno-miR-466c-5p expression was upregulated, and Foxo4, Pax1, Nkx3-2, and Sox9 somatic cell–associated genes were downregulated in VAD-CS embryos. Specifically, rno-miR-466c-5p could bind to the 3′-end untranslated region (3′-UTR) of Foxo4, thereby downregulating Foxo4 levels. Luciferase and siRNA assays revealed that SULT1C2A downregulates rno-miR-466c-5p expression through the PI3K-ATK signaling pathway, thereby increasing Foxo4 expression. Foxo4 is a forkhead box (Fox) transcription factor that participates in the development of cartilage and skeletal muscle during embryonic development (Matsuzaki et al., 2018). Therefore, it was suggested that increasing the expression of lncRNA SULT1C2A may be a possible treatment option for VAD-CS.

### miRNA

miRNA is a small type of RNA that can bind to mRNA to promote its expression or breakdown. Overall, miRNA contributes to the development of scoliosis through the action of signaling pathways. In 2017, Jiang Heng et al. selected muscle tissue from the convex and concave sides of the paravertebral vertebrae of three female patients with AIS (Jiang, 2017); corresponding RNA sequencing analysis identified a total of 18 differentially expressed miRNAs, with the convex side significantly overexpressing miR-517a-3p and the concave side overexpressing miR-424-3p. These two miRNAs are located on the mammalian miRNA gene cluster Gtl2-Dio3 and regulated by the transcription factor MEF2A. Therefore, it was concluded that these miRNAs act primarily through the Wnt signaling pathway and are associated with skeletal muscle regeneration, fibrosis, and cartilage development, which, in turn, contribute to paravertebral muscle imbalance and influence the onset and progression of AIS. Hui et al. also analyzed the differential miRNA expression observed in the BMCs of patients with AIS and normal subjects (Hui et al., 2019); a total of 54 previously unreported differential miRNAs were identified, with key functions in small GTPase-mediated signal transduction, DNA-dependent transcription, cytoplasmic division, cell adhesion, transmembrane transport, and hypoxic responses. Pathway analysis demonstrated that differentially expressed miRNAs were primarily concentrated in the MAPK, PI3K-Akt, calcium, Notch, and ubiquitin-mediated protein hydrolysis pathways. These pathways play an integral role in osteogenesis and adipose differentiation (Kanno et al., 2007; Baker et al., 2015), which are involved in the development of AIS. In this study, the seven differentially expressed miRNAs, including miR-17-5p, miR-106a-5p, miR-106b-5p, miR-16-5p, miR-93-5p, miR-15a-5p, and miR-181b-5p, were most correlated with the development of AIS and significantly associated with concomitant bone loss.

The differences of non-coding RNA changes in the bone tissue samples could better illustrate scoliosis pathogenesis. Zhang et al. analyzed miRNA microarrays in iliac bone tissue from patients with AIS and normal controls and identified miR-145 as a miRNA associated with AIS (Zhang et al., 2018). RT-qPCR confirmed that miR-145 was aberrantly upregulated in patients with AIS; additionally, aberrantly high expression of this miRNA was also found in bone cells from patients with AIS cultured *in vitro*. miR-145 expression was positively correlated with the expression of *CTNNB1*, which encodes β-catenin; further, continued activation of β-catenin may impair osteoblast function in patients with AIS. After knocking down the miR-145 gene in AIS osteoblasts, miR-145 expression was downregulated, the formation of an active β-catenin/Tcf4 complex was significantly inhibited, and normal expression of osteoblast markers, including osteoprotegerin (OPG), transmembrane glycoprotein E11, and sclerostin secretion (SOST), was restored. Additional specific disorders can accompany scoliosis. In 2018, Seco-Cervera et al. analyzed miRNA differences between patients with Friedreich’s ataxia (FRDA) and controls (Seco-Cervera et al., 2018). FRDA is a rare genetic disorder that causes progressive neurological impairment and motor problems; patients with FRDA often have scoliosis, heart disease, and diabetic complications (Filla et al., 1996). hsa-miR-128-3p, hsa-miR-625-3p, hsa-miR-130b-5p, hsa-miR-151a-5p, hsa-miR-330-3p, hsa-miR-323a-3p, and hsa-miR-142-3p were the most significant differentially expressed miRNAs, with these correlation results being validated by RT-qPCR. Of these, hsa-miR323a-3p could be used as a biomarker to discriminate patients with or without cardiac disease. However, this study did not further explore the interactions between these miRNAs and the corresponding mRNAs or the possible mechanisms influencing disease onset or progression.

The potential of miRNAs as biomarkers for identifying scoliosis is extensive. In 2020, Wang et al. evaluated five patients with mild AIS (Cobb angle >40°), five patients with severe AIS (Cobb angle >50°), and five healthy controls and sequenced their serum samples for miRNA (Wang et al., 2020). Corresponding results revealed that miR-941, miR-151a-3p, and miR-148b-5p were significantly elevated in the serum of the patients with AIS. When evaluating patients in the validation cohort, miR-151a-3p was determined to be significantly elevated in the sera of patients with severe scoliosis. After analysis of miRNA target genes, they ultimately established that *GREM1* expression levels were significantly downregulated in osteoblasts from patients with severe AIS, thus indicating that miR-151a-3p may affect bone homeostasis and contribute to the development of AIS by binding to *GREM1*. miR-151a-3p could be used as a biomarker to predict severe AIS. In 2022, Chen et al. evaluated four patients with severe AIS and four age-matched controls (Chen et al., 2022); in this study, they extracted intraoperative iliac spine bone tissue for RNA microarray analysis. miR-96-5p expression was significantly higher in patients with AIS. An AIS prediction model combining serum miR-96-5p levels, patient age, age at menarche, and body weight was developed; an area under the ROC curve of 0.752 without the need for a bone mass scan demonstrated the corresponding potential of miR-96-5p as a biomarker for AIS.

Some studies have also used animal models to explore the role of miRNAs in scoliosis. Ishiwata et al. determined that miR-224-5p expression was greatly upregulated in the lumbar spine of a CS model (Ishibashi rats) compared to that in normal 42-day-old rats (Ishiwata et al., 2020). Further, a concomitant upregulation of miR-224-5p-regulated fibrinogen inhibitor-1 expression and an increase in type I collagen expression were also observed in this CS model. Overall, this finding indicated high osteoblast differentiation in the CS rat model. Further target gene analysis of miR-224-5p was not performed in this study; nonetheless, these results show that miR-224-5p can bind to target genes and contribute to the development of CS by affecting osteoblast differentiation. There are various effects of air pollution on embryonic congenital development (Choe et al., 2018), with the occurrence of CS being potentially closely linked to air pollution (Li et al., 2015). In 2019, Li et al. divided pregnant rats into randomized experimental and control groups, which were placed into environments with differing air qualities, indicated by PM2.5 (particulate matter with particles less than 2.5 μm in diameter) concentrations (Li Z. et al., 2019); specifically, experimental rats were placed in an environment with a PM2.5 > 200 μg/m³ and control rats were housed in an environment with a PM2.5 < 50 μg/m³. Embryos were removed for RNA sequencing on day 9 of pregnancy, and a total of 291 differentially expressed miRNAs were identified between the two groups, of which 204 miRNAs were upregulated and 87 miRNAs were downregulated in the experimental group. Convergent analysis suggested that these miRNAs were primarily involved in mitotic spindle organization, cellular respiration, ethanoic acid metabolism, and the proteasome, ultimately contributing to the development of CS.

### circRNA

In recent years, the possibility of utilizing specific circRNAs in the serum as diagnostic biomarkers for scoliosis-related diseases has been reported. García-Giménez et al. reported differences in the distribution of circRNA abundance between patients with AIS and normal controls (Garcia-Gimenez et al., 2018). They found that the abundance of three circRNAs, miR-122-5p, miR-27a-5p, and miR-223-5p, was significantly higher in patients with AIS than those in the normal population. These three circRNAs, together with miR-1306-3p, could be used as biomarkers to distinguish patients with AIS from normal controls. This method was validated in 30 patients with AIS compared to 13 normal subjects, with a corresponding area under the ROC curve of 0.95 and a high sensitivity, demonstrating the potential of using circRNA in combination with miRNA for AIS diagnosis. Further, Liu et al. identified a total of 22 differentially expressed circRNAs in patients with CS compared to controls (Liu et al., 2020); further, seven of these circRNAs in the validation group were confirmed by qPCR. Among them, hsa_circ_0006719 levels were highly elevated in the peripheral blood of patients with CS, and a prediction model based on this revealed an area under the ROC curve of 0.73, indicating a strong potential for the use of hsa_circ_0006719 as a diagnostic biomarker for CS.

Some studies have also focused on the overall variation of the three types of non-coding RNAs in patients with scoliosis. In 2018, Chen et al. sequenced non-coding RNAs with mRNAs in VAD-CS rat embryos (Chen et al., 2018). A total of 56 miRNAs, 70 circRNAs, and 685 lncRNAs were observed to be differentially expressed in VAD-CS and normal rat embryos. Some non-coding RNAs were further validated by RT-qPCR (miRNAs: miR-187-5p, miR-466c-3p; lncRNAs: NONRATG027649.1, NONRATG024332.1; circRNAs: chr5_50556456_51183813, chr15_23792823_23793342). Additionally, enrichment analysis demonstrated that these differentially expressed RNAs functioned in Foxo, PI3K-Akt, mTOR, EGFR, Wnt, and other signaling pathways.

In 2020, Li et al. searched PubMed, EMBASE, and GEO databases for literature that compared differences in gene, miRNA, and lncRNA expression between patients with AIS and normal control MSCs (Li et al., 2020); a computerized pooled analysis was then performed to establish a network of mRNA–miRNA–lncRNA interactions. A total of 1,027 DEGs (551 upregulated, 476 downregulated), 54 differentially expressed miRNAs (42 upregulated, 12 downregulated), and 658 differentially expressed lncRNAs (345 upregulated, 313 downregulated) were included in this review. Overall, a total of 6 mRNA–miRNA–lncRNA action networks were observed: RAP2C–AS1–miR-4419b–WDTC1, TSPEAR–AS2–miR-16-5p–CYCS/KDR/PDIA6/TGOLN2/HSPA5, TSPEAR–AS2/HCG18–miR-93-3p–CYCS/HSPA5, TSPEAR–AS2–AS2/HCG18–miR-93-5p–CYCS/TGOLN2, TSPEAR–AS2/HCG18–miR-615-3p–CKAP4 and TSPEAR–AS2/HCG18–miR-125a-3p–PGK1. These networks suggest that the non-coding RNAs may form their own reticulated regulatory system; however, these axes of action have only been analyzed in this study, and their accuracy needs to be confirmed by further experiments (Table 2).

### Histone modification

Histone modification such as methylation and acetylation is an epigenetic modification that affects transcriptional activity (Delcuve et al., 2009). Mao et al. genotyped 500 patients with AIS and 494 age-matched controls using PCR-based Invader analysis (Mao et al., 2013). These results demonstrated that rs12459350, which regulates histone lysine 79 (H3K79) methylation, was strongly correlated with AIS susceptibility; however, this study did not further explore the specific mechanisms in this process.

In 2019, Li et al. performed histological and genetic testing of articular cartilage from 11 patients with IS compared to 10 matched controls (Li J. et al., 2019). Light microscopy of hematoxylin and eosin-stained samples revealed a significant increase in particular chondrocyte proliferation but a reduced articular cartilage thickness in the IS patient samples compared to those of the controls, suggesting that abnormal hyperplasia occurred in the samples taken from the patients with IS. Further examination revealed that the patients with IS exhibited increased expression of type II collagen and Bcl2 protein, which promote chondrocyte proliferation; therefore, it was concluded that the increased expression of Bcl2 was directly responsible for the abnormal cartilage proliferation. Examination of miRNA expression in articular cartilage tissue showed that patients had downregulated expression of a miRNA (miR-15a) that inhibits Bcl2, increased expression of the methyltransferase SUV39H1, and increased trimethylation of the miR-15a promoter region with histone H3K9. Thus, histone methylation may cause abnormal proliferation of chondrocytes through the miR-15a/Bcl2 signal axis, ultimately inducing abnormal spinal growth and leading to the development of scoliosis.

## Chromatin remodeling

Chromosomes consist of nucleosomal units of DNA bound to histones, which enables the genome to be highly condensed (Clapier and Cairns, 2009). Chromatin remodeling complexes (remodelers) reorganize or disrupt nucleosomes through the energy released by ATP and are required for transcriptional expression, DNA replication, and DNA repair; further chromatin remodeling is largely dependent on these processes conferred by remodelers (Clapier and Cairns, 2009). This mechanism protects genes when important gene fragments are not functioning and controls the appropriate length of gene fragment exposure during replication and transcription (Clapier and Cairns, 2009). Chromatin reformulation is one of the key epigenetic mechanisms that control gene expression and has been implicated in a number of diseases, such as cerebro-oculo-facial-skeletal syndrome (Citterio et al., 2000) and Williams-Beuren syndrome (Kitagawa et al., 2003), alongside tumorigenesis and cell invasion behaviors in cancer (Denslow and Wade, 2007). However, no prior studies have reported the impact of chromatin remodeling on the pathogenesis of scoliosis; therefore, we hope that future studies explore this area further to provide a new understanding of epigenetics in scoliosis.

## Conclusion

Epigenetic regulation plays an essential role in the pathogenesis of scoliosis. Consequently, recent studies have explored the relationship between scoliosis and epigenetics, with primary focus on two mechanisms: DNA methylation and non-coding RNA regulation. These mechanisms have specifically been evaluated by analyzing the changes in the expression of key products and their corresponding influence on signaling pathways, leading to a deeper understanding of the pathogenesis of scoliosis. However, the small number of studies that can be cross-examined may affect the corresponding depth of understanding within this review. Most studies analyzing epigenetics in scoliosis have not yet adopted methods such as downstream protein analysis to validate their proposed theories of pathway effects; therefore, there remains the requirement for further studies to deepen the understanding of DNA methylation and non-coding RNA regulation in scoliosis by testing the validity of current studies and further evaluating the mechanisms involved in this pathogenesis. Further, there are few studies on histone modification and chromatin remodeling, and the effects of chromatin remodeling on scoliosis are yet to be elucidated. Because the process of chromatin remodeling can be affected by a range of cellular behaviors, such as replication, transcription, and repair, the corresponding research is less reliable; therefore, developing an understanding of chromosome remodeling patterns specific to patients with scoliosis will likely be challenging. To conclude, epigenetic research remains an emerging field in scoliosis, and further studies are required to improve our current understanding of the pathogenesis of scoliosis and provide new insights into the future clinical diagnosis and treatment of this disorder.
